# Anesthesia, sex and miscarriage history may influence the association between cesarean delivery and autism spectrum disorder

**DOI:** 10.1186/s12887-021-02518-1

**Published:** 2021-02-01

**Authors:** Ye Yang, Jingjing Lin, Xiaozi Lu, Guanglei Xun, Renrong Wu, Yamin Li, Jianjun Ou, Yidong Shen, Kun Xia, Jingping Zhao

**Affiliations:** 1 National Clinical Research Center for Mental Disorders, Department of Psychiatry, and China National Technology Institute on Mental Disorders, The Second Xiangya Hospital of Central South University, Changsha, 410011 Hunan China; 2 Qingdao Mental Health Center, Qingdao, 266034 Shangdong China; 3 Shandong Mental Health Center, 49 East Wenhua Road, Jinan, 250014 Shandong China; 4 Clinical Nursing Teaching and Research Section, The Second Xiangya Hospital, Central South University, Changsha, 410011 Hunan China; 5 Center for Medical Genetics and School of Life Sciences, Central South University, Changsha, 410078 Hunan China

**Keywords:** Autism spectrum disorder, Cesarean section, Anesthesia, Sex, Miscarriage history

## Abstract

**Background:**

To explore the association between cesarean section (CS) and risk of autism spectrum disorder (ASD), and evaluate the possible factors influencing this association.

**Methods:**

In total, 950 patients diagnosed with ASD and 764 healthy controls were recruited in this study. Socio-demographic characteristics and prenatal, perinatal, and neonatal characteristics were compared between the two groups. Univariate and multivariable conditional logistic regression analyses were applied to adjust for confounders. Further stratified analyses based on sex and miscarriage history were similarly performed to explore the factors influencing the association between CS and ASD.

**Results:**

CS was evidently associated with an elevated risk of ASD (adjusted odds ratio [aOR] = 1.606, 95% confidence interval (CI) = 1.311–1.969). Unlike regional anesthesia (RA), only CS performed under general anesthesia (GA) consistently elevated the risk of ASD (aOR = 1.887, 95% CI = 1.273–2.798) in females and males in further stratified analysis. The risk of children suffering from ASD following emergency CS was apparently increased in males (aOR = 2.390, 95% CI = 1.392–5.207), whereas a higher risk of ASD was observed among voluntary CS and indicated CS subgroups (aOR = 2.167, 95% CI = 1.094–4.291; aOR = 2.919, 95% CI = 1.789–4.765, respectively) in females. Moreover, the interaction term of CS and past miscarriage history (*β* = − 0.68, Wald χ2 = 7.5, df = 1, *p* = 0.006)) was similarly defined as influencing ASD.

**Conclusions:**

The exposure of children to GA during CS may explain the possible/emerging association between CS and ASD. In addition, sex and miscarriage history could equally be factors influencing the association between CS and ASD.

## Background

Autism spectrum disorder (ASD) is a neurodevelopmental disorder in which the exact mechanism remains unclear and is characterized by impairment in social communication, alongside restricted interest and repetitive behaviors [1]. ASD has been of general concern globally, with a substantial increase in prevalence in recent decades [2]. Although ASD is highly genetic [3], there is evidence suggesting the involvement of numerous prenatal and perinatal factors in its development [3–7]. Among all gestational and obstetric risk factors of ASD, accumulated evidence suggests an association between cesarean section (CS) and ASD which is worrisome given the dramatically increasing trend in CS implementation in numerous countries [8, 9]. A recent meta-analysis demonstrated that a 23% increase in the risk of ASD was associated with CS compared to vaginal delivery [10]. Even though previous studies have reported that CS is not a risk factor for ASD [11, 12], most studies have reached a consistent conclusion [13, 14]. The incidence of CS is increasing globally, with an evaluated annual increase of 4% [15]. Surprisingly, the incidence of CS has increased rapidly in China over the past decade, with a current national rate of approximately 40% [16]. It is becoming increasingly important to fully understand the association between CS and ASD.

The CS is a surgical procedure of delivering a baby to prevent a risk to the health of the mother or baby accompanied by vaginal delivery. Emergency CS is usually performed in an urgent situation due to complications, such as fetal distress which could occur spontaneously [17]. On the contrary, indicated CS (planned or elective CS) is scheduled beforehand and is mostly necessary for obstetric or medical indications such as multiple gestations, suspected macrosomia, or pre-existing obstetric conditions [18]. Nevertheless, in some countries, CS is implemented in a minority of the women, only on maternal request without medical indications (voluntary CS) [19]. Although CS is a relatively safe method compared to vaginal delivery, it has previously been linked to adverse health outcomes in newborns [20–22]. CS may directly and indirectly affect neurodevelopment, as well as cognitive outcomes [23]. Indirect influence could occur via established correlations between CS and negative outcomes on child health, that is associated with neurodevelopment, including disturbed gut microbiota and changes in stress response, among others [24, 25]. The existing evidence suggests that there is a significant correlation between CS and neurodevelopmental disorders, including ASD and attention deficit hyperactivity disorder (ADHD) [10, 26].

Although the association between CS and ASD has been established in several previous studies, the effect size is heterogeneous when allowing for the adjustment for different confounding factors [27, 28]. Data from previous studies have reported the risks of ASD in children following indicated CS and emergency CS were nearly identical compared with vaginal delivery; however, voluntary CS was not included by existing studies [14, 29]. Contradictory research has suggested that indicated and emergency CS are related to different indications and thus may be associated with differential neurodevelopment process [30]. Alternatively, the observed association may be due to the inclusion of more complete types of surgery indications in the analysis, considering that other confounding factors were controlled.

Emerging evidence has suggested an association between the types of anesthesia and risk of ASD development [14, 31]. CSs are usually performed under general anesthesia (GA) or regional anesthesia (RA); however, RA is more frequently used because GA increases the complications of CS [32, 33]. Exposing the premature brains of the fetuses to obstetric anesthesia can result in histopathological damage, which can affect brain development [34, 35]. The exposure of mothers to GA during labor was significantly associated with higher risk of ASD [14, 31]. On the contrary, inconsistent results have shown that the early exposure of children to anesthesia in the uterus, first 2 years of life, or afterwards is not associated with a higher risk of ASD [36]. Given the contradiction among the conclusions of various research, the susceptibility of a fetus to ASD subjected to obstetric anesthesia during CS requires further exploration.

There could be variation in the observed correlation based on concomitant confounding factors or confounding by indications; this indicates that ASD may be related to the indications and concomitant confounding factors of ASD, rather than the surgical procedure [10]. Considering that different types of CS are accompanied by heterogeneous and complex confounding factors, understanding the risk of ASD among different types of CS is crucial for the early detection and prevention of ASD. Therefore, this study aimed to explore the correlation between different types of CS and ASD, while taking various prenatal and perinatal confounders into account, based on data from a large retrospective case-control study that included comprehensive clinical data on the pregnancy and birth of these children.

## Methods

### Subjects

This study was conducted following the recommended guidelines prescribed by the Second Xiangya Hospital Ethics Committee. All subjects gave written informed consent following the Declaration of Helsinki. In this case-control study, both cases and controls were ascertained from two-stage data collections from training centers and special education schools across the country. The first and second stage recruitment of subjects and data collection were performed from 2012 to 2016 and 2016–2018, respectively. The data collection was conducted in two stages because scale entries were further refined and more detailed gestational and obstetric confounding factors were included in the second stage. Subjects in the first stage who were diagnosed with Autistic Disorder or Pervasive Developmental Disorder - Not otherwise specified (PDD-NOS) in accordance with the criteria established in the Diagnostic and Statistical Manual of Mental Disorders Fourth Edition (DSM- IV) were eligible for this study. These diseases, as mentioned earlier, fulfilled the diagnostic criteria of DSM-5 for ASD. For subjects in the second stage, the diagnostic criteria of the Diagnostic and Statistical Manual of Mental Disorders Fourth Edition (DSM-5) was employed. Children with ASD following the criteria set out in DSM-5 were enrolled in this study. ASD was diagnosed by two experienced pediatric psychiatrists, according to DSM- IV or DSM- 5 criteria.

Children with severe physical diseases such as congenital heart disease or hematological diseases, or the first diagnosis is other psychiatric disorders, such as schizophrenia or Intellectual Disability, were excluded. Autistic patients with chromosomal abnormalities, such as Fragile X syndrome and Down’s syndrome, were equally excluded. Since previous studies have shown that gestational age may be a significant influencing factor of ASD, newborns of gestational age <37 or >42 weeks were excluded from this study. Similarly, patients who underwent assisted delivery, including the use of forceps, vacuum, and oxytocin were excluded from this study.

**Fig. 1:**
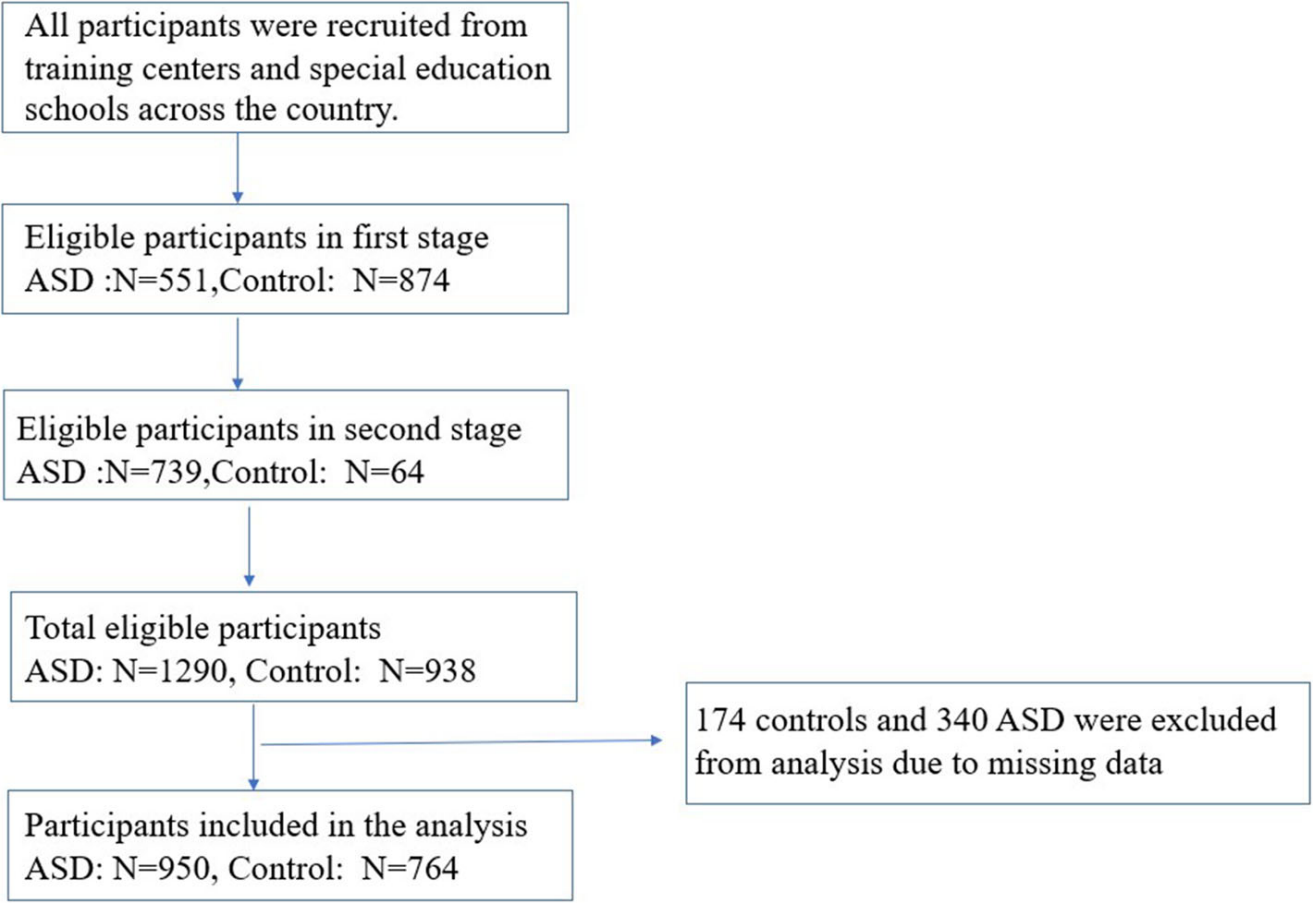
Flowchart of participation in the study

Controls were recruited from neighborhood kindergartens and in the same city. Typically developing children were recruited by adapting a survey questionnaire that include basic information and a screening test for preliminary screening of neurological or neurodevelopment disorder. Other exclusion criteria are similar to those in the autism group. Five hundred fifty-one patients and 874 healthy controls were recruited in the first stage, while 739 patients and 64 healthy controls were recruited in the second stage. In total, 1290 patients and 938 healthy controls were recruited in current study. However, 174 controls and 340 children with autism were excluded from analysis due to missing data. Ultimately, 950 children with autism and 764 controls were enrolled for the subsequent data analyses, the research process is shown in Fig. 1.

### Data collection

Details of data collection have been described in a previously published study [37]. In summary, data on the subjects were collected from a general socio-demographic questionnaire administered to the parents/guardians of all ASD cases and controls during enrollment. In addition to demographic, clinical, and behavioral data of the subjects, prenatal, perinatal and neonatal variables for both cases and controls were collected in this study. Behavioral data were evaluated by Social Responsiveness Scale (SRS). Prenatal characteristics included parental age, maternal childbearing age, and past miscarriage history. Perinatal characteristics of the enrolled children included parity number, gravity number, past miscarriage history, threatened abortion, mode of delivery (CS vs. vaginal delivery), surgery indication (emergency CS, voluntary CS and indicated CS) and types of anesthesia (RA vs. GA), which were considered as independent variables for this analysis. Infant variables for both cases and controls included sex, age and birth weight.

### Statistical analysis

Data analyses were performed using SPSS version 18 (Armonk, NY, USA). The socio-demographic characteristics, as well as the correlations of CS and maternal, perinatal, and neonatal characteristics with the risk of ASD, were assessed via appropriate bivariate statistical tests. In the event data did not follow a normal distribution, we used a Mann-Whitney test. We used a *t-*test when the data were normally distributed. In addition, categorical variables were compared using the chi-square or Fisher exact tests. The univariate conditional logistic regression models were used in the assessment of the univariable association of various prenatal, perinatal and neonatal characteristics with ASD. Further multivariate conditional logistic regression models were conducted to evaluate the adjusted effect of CS on the risk of ASD (compared to vaginal deliveries). The variables that were obviously correlated with ASD (*p*-value < 0.05) were considered potential confounders and included in this model. Subgroup analyses were performed to assess the influence of various CS types on the risk of ASD. CS deliveries were stratified to different types according to the type of anesthesia and the indications for CS. The associations were analyzed by sex separately to evaluate the sex-specific relationship between CS and ASD and to remove the effects of an unbalanced sex ratio. Similarly, the analysis was separately conducted according to the history of miscarriage because it is a significant risk factor for ASD in current studies [38].

## Results

In total, 950 ASD cases and 764 subjects (as controls) were included in this study. The median age of the ASD cases and controls were 4.0 and 4.8 years, respectively. Majority of the ASD cases were male (792/158), whereas the sex distribution in the controls was balanced (407/357). Table 1 shows the association between prenatal, perinatal, and infant characteristics and ASD risk, respectively. CS was correlated with the risk of ASD (*p* <0.001). Apart from CS, other variables that were associated with the risk of ASD included: parity number (*p* <0.001), history of miscarriage (*p* <0.001) and infant’s weight at birth (*p* = 0.001). Behavioral characteristics of the samples are presented in Table 2.

**Table 1:** Prenatal, perinatal, and infant characteristics of cases and control subjects

Variables	ASD (*N* = 950)	Control (*N* = 764)	*P*
Maternal and prenatal characteristics, N (%)			
Mother’s age at birth (Years)	28.0 (25.9, 30.0)	28.0 (25.0, 30.3)	0.812
Parity number	1 (1.2)	1 (1.2)	**<0.001**
Gravity number	1 (1.1)	1 (1.1)	0.828
Past miscarriage history	315 (34.4)	119 (18.1)	**<0.001**
Threatened abortion	127 (13.6)	79 (10.6)	0.072
Labor and perinatal characteristics, N (%)			
Cesarean section, Infant characteristics	491 (51.7)	301 (39.4)	**<0.001**
Infant’s weight at birth (kg), Median (IQR)	3.5 (3.2,3.9)	3.4 (3.1,3.8)	**0.001**

Bold values are statistically significant associations (*p*-value < 0.05)

*ASD* Autism spectrum disorder

Values are presented as median (IQR [interquartile range])

**Table 2:** Behavioral characteristics of parents and children

SRS	ASD (*N* = 950)	Control (*N* = 764)
Total score, mean ± SD (min, max)	95.0 ± 25.1 (20.170)	51.9 ± 18.1 (1,84)
Social awareness, mean ± SD (min, max)	13.6 ± 3.6 (1,24)	5.0 ± 2.7 (0,17)
Social cognition, mean ± SD (min, max)	18.8 ± 5.1 (0,33)	10.0 ± 4.0 (0,16)
Social communication, mean ± SD (min, max)	32.7 ± 9.3 (9,61)	16.3 ± 6.9 (0,48)
Social motivation, mean ± SD (min, max)	15.8 ± 4.9 (1,31)	9.3 ± 3.8 (0,17)
Autistic mannerisms, mean ± SD (min, max)	14.1 ± 6.6 (1,35)	6.2 ± 4.8 (0,7)

*SRS* Social Responsiveness Scale

Univariate conditional logistic regression analyses revealed that CS was significantly associated with ASD (adjusted odds ratio [aOR] = 1.606; 95% confidence interval [CI] = 1.311–1.969). This association was equally observed between CS and the parity number, and past history of miscarriage. Further multivariate conditional logistic regression models revealed an association between the risk of CS delivery (vs. vaginal delivery) and ASD. After adjusting for prenatal, perinatal or neonatal confounding factors that were associated with ASD (*p* < 0.05) in this study (Table 3), the results similarly indicated a significant association between CS and ASD (aOR = 1.573; 95% CI = 1.241–1.992). While parity number was not a risk factor for ASD as demonstrated by univariate conditional logistic regression analyses, multiple regression analyses interestingly identified the interaction term of CS and history of miscarriage (*β* = − 0.68, Wald χ2 = 7.5, df = 1, *p* = 0.006) as an influencing factor for ASD.

**Table 3:** Univariate and multivariate analyses for the risk of ASD

Variable	Univariate		Multivariate	
	Adjusted OR (95% CI)	*P*-value	Adjusted OR (95% CI)	*P*-value
Parity number	1.265 (1.114–1.436)	**<0.001**	0.930 (0.776–1.115)	0.434
Past miscarriage history	2.069 (1.579–2.679)	**<0.001**	2.262 (1.501–3.410)	**<0.001**
Cesarean section	1.606 (1.311–1.969)	**<0.001**	1.573 (1.241–1.992)	**<0.001**
Infant’s weight at birth	0.989 (0.968–1.010)	0.079	1.001 (1.000–1.001)	**0.026**

Bold values are statistically significant associations (*p*-value < 0.05)

*OR* Odds ratio, *CI* Confidence intervals

### Subgroup analysis

**Fig. 2:**
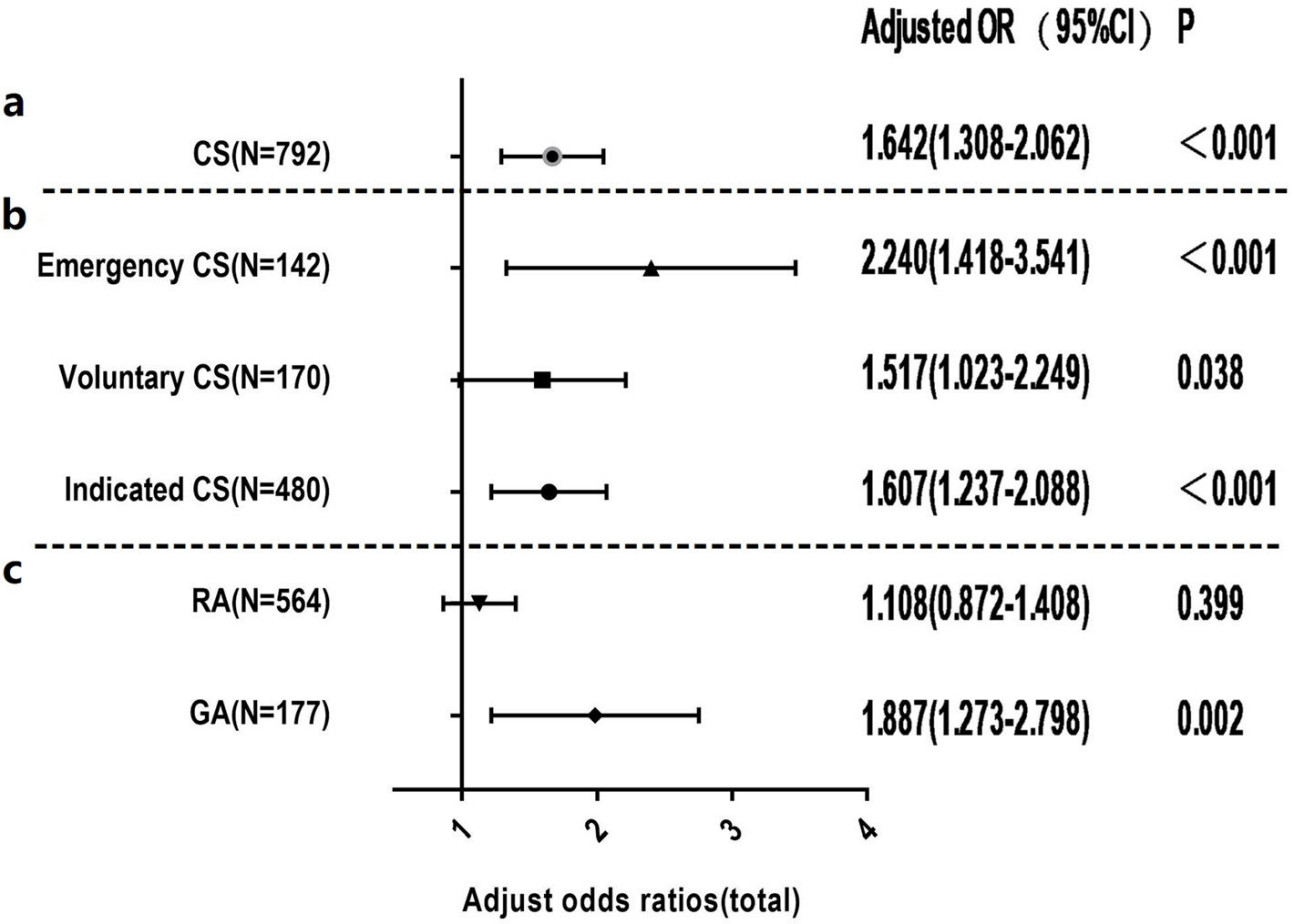
**a** A forest plot of the association between cesarean section (CS) and Autism Spectrum Disorder (ASD) in total study samples; **b** Adjusted odds ratios and two-sided 95% confidence intervals of ASD following CS compared with vaginal delivery in different anesthesia regimen subgroups (regional anesthesia [RA] vs. general anesthesia [GA]); **c** Adjusted odds ratios and two-sided 95% confidence intervals of ASD following CS compared with vaginal delivery in different surgery indication subgroups (emergency CS, voluntary CS and indicated CS). Gray dashed horizontal lines separate the results of different stratification analyses. Adjusted odds ratio for CS was estimated from logistic regression after adjusting for confounding factors (e.g., parity number, past miscarriage history, subject’s sex and age, and past miscarriage history*CS). Adjusted odds ratio for different surgery indication was adjusted for confounding factors (parity number, past miscarriage history, children’s sex and age). Adjusted odds ratio for anesthesia regimen was adjusted for confounding factors (parity number, past miscarriage history, infant’s weight and children’s sex and age)

In order to further explore the mechanism underlying the association between CS and ASD, the sample was stratified according to different surgery indications and types of anesthesia; the same conditional logistic regression models were applied in these subgroups (Fig. 2). After adjusting for potential risks that were correlated with ASD, (Table 3), it was observed in the stratified analysis that a similar risk exists for ASD between voluntary CS and indicated CS (aOR = 1.517, 95% CI = 1.023–2.249; aOR = 1.607, 95% CI = 1.237–2.088 respectively). On the contrary, emergency CS was associated with a higher risk than voluntary CS and indicated CS compared to vaginal deliveries (aOR = 2.240, 95% CI = 1.418–3.541). Subsequently, CS was stratified according to the anesthesia regimen, and 51 subjects with no record of anesthesia were excluded from the analysis. This model revealed that CS performed under GA indicated a 1.8-fold increased risk of ASD compared with vaginal deliveries (aOR = 1.887; 95% CI = 1.273–2.798). However, insignificant differences in the risk of ASD were observed between neonates delivered by CS under RA and those delivered vaginally (*p*>0.05).

### Sex stratification analysis

**Fig. 3:**
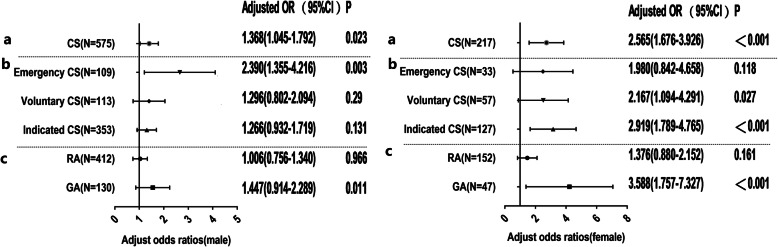
Adjusted odds ratios and two-sided 95% confidence intervals of Autism Spectrum Disorder (ASD) following cesarean section (CS) compared with vaginal delivery (VD) for further stratified analysis of sex. The left and right present male and female results, respectively. **a** Forest plots of the association between CS and ASD in male and female study samples. The analysis was further classified based on methods of anesthesia (RA vs. GA) and surgery indication subgroups (emergency CS, voluntary CS and indicated CS), presented in **b** and **c**, respectively. Gray dashed horizontal lines separate the results of different stratification analyses. Adjusted odds ratio for CS was estimated from logistic regression after adjusting for confounding factors (e.g., parity number, past miscarriage history, children’s age, and past miscarriage history*CS). Adjusted odds ratio for different surgery indication was adjusted for confounding factors (e.g., parity number, past miscarriage history, subject’s age). Adjusted odds ratio for anesthesia regimen was adjusted for confounding factors (e.g., parity number, past miscarriage history and infant’s weight, and children’s age)

Given the unbalanced sex distribution in the ASD cases, separate analyses were conducted according to sex (Fig. 3), after controlling for potential risks. The risk of ASD among neonates delivered by CS compared with those delivered vaginally was approximately twice higher in females than males (aOR = 2.565, 95% CI = 1.676–3.926; aOR = 1.368, 95% CI = 1.045–1.792, respectively). In males, the aOR of ASD following CS was elevated across the emergency subgroups (aOR = 2.390, 95% CI = 1.392–5.207). In addition, no risk of ASD was observed among voluntary CS and indicated CS (both *p*>0.05). The children delivered by CS under GA demonstrated a 44% higher risk of developing ASD than those delivered vaginally (aOR = 1.447, 95% CI = 0.914–2.289). Notably, this model indicated no statistical difference in the risk of ASD between children delivered by CS under RA and those delivered vaginally (*p* = 0.966).

Unlike males, a higher risk of ASD was observed among the voluntary CS and indicated CS subgroups (aOR = 2.167; 95% CI = 1.094–4.291; aOR = 2.919; 95% CI = 1.789–4.765, respectively) in females. However, the analysis revealed no significant difference in the risk of developing ASD in the emergency CS subgroup compared to vaginal deliveries subgroup (*p>*0.05). Similarly, an increased risk of ASD was correlated with CS under GA (aOR = 3.588, 95% CI = 1.757–7.327), unlike CS under RA(*p* >0.05), after adjusting for potential risks.

### Stratification analysis relative to miscarriage history

**Fig. 4:**
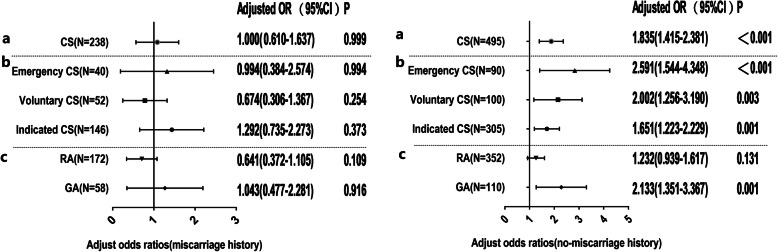
Adjusted odds ratios and two-sided 95% confidence intervals of Autism Spectrum Disorder (ASD) following cesarean section (CS) compared with vaginal delivery (VD) for further analysis of past miscarriage history. The left and right present the results of analysis with and without a history of miscarriage, respectively. **a** Forest plots of the association between CS and ASD in study samples with and without a history of miscarriage. The analysis was further classified based on methods of anesthesia (RA vs. GA) and different surgery indication subgroups (emergency CS, voluntary CS and indicated CS), presented in **b** and **c**, respectively. Gray dashed horizontal lines separate the results of different stratification analyses. Each adjusted odds ratio was estimated from logistic regression after adjusting for confounding factors (parity number, gestational diabetes, infant’s weight and children’s sex, and age)

Due to the interaction between miscarriage history and CS in the risk of developing ASD, further stratified analysis, according to miscarriage history, was similarly performed in this study (Fig. 4). Fifty-nine subjects with no record of miscarriage history were excluded in the analysis. Interestingly, CS was not significantly associated with the risk of ASD among subjects with a history of miscarriage (*p* = 0.999). In addition, further subgroup analysis equally revealed no significant association between different types of CS and ASD (all *p* >0.05). In line with the results above, CS is a significant risk factor for ASD in subjects without a history of miscarriage (aOR = 1.835, 95% CI = 1.415–2.381). In addition, the OR of ASD risk following emergency CS (aOR = 2.591, 95% CI = 1.544–4.348) versus vaginal delivery was obviously higher than indicated CS (aOR = 1.651, 95% CI = 1.223–2.229) and slightly higher than voluntary CS (aOR = 2.002, 95% CI = 1.256–3.190). The neonates delivered by CS performed under GA had approximately two times higher risk of suffering from ASD than those birthed by vaginal delivery (aOR = 2.133, 95% CI = 351–3.367). Similarly, no association was observed between ASD and CS performed under RA (*p* >0).

## Discussion

This study demonstrated that the correlation between CS and ASD is exclusive to CSs performed under GA, with an evident heterogeneity in different surgical indications and sex. In addition, CS may interact with miscarriage history to influence the development of ASD.

According to this study, delivery by CS was associated with a higher risk of ASD in children, even after adjusting the potential confounding factors. The result was supported by previous well-established observations that increased risk of ASD was associated with delivery by CS [13, 27, 29]. Further sex stratification analysis revealed a sex-biased ratio in the risk of developing ASD among births by CS, irrespective of the different surgical indications and method of anesthesia. The risk of ASD among females which was twice higher in children delivered by CS than in those delivered vaginally was consistent with several previous reports [14, 31]. Evidently, females were more sensitive to the long-term effects of CS than males. Therefore, further research is required to investigate the mechanisms underlying the observed sex-related distinctions in the risk of ASD among births by CS.

The results supported the original hypotheses that emergency CS is associated with a higher risk of ASD than voluntary CS and indicated CS, and the risk of ASD following indicated CS is slightly higher than the risk following voluntary CS. Consistent results were observed in previous studies that both indicated CS (elective CS or planned CS) and emergency CS were associated with an increased risk of ASD, contrary to this study [14, 29, 31]. These discrepancies may be attributed to the heterogeneity of subgroups relative to surgical indications employed in different studies, contrary to this study. Previous analysis included emergency CS and indicated CS (elective CS or planned CS), excluding voluntary CS. Considering this result, emergency CS may confer additional risk of ASD beyond risks associated with factors leading to CS among indicated CS and voluntary CS. This indicates that CS may exclusively exert an inherent risk on ASD, and there might be an induced risk from complex rather than unequal confounding factors from different CS surgical indications (not CS exclusively). Existing evidence supports these hypotheses; CS, whether planned or emergency, appears to be correlated with a complex mix of confounding factors equally related to ASD risk [39]. A sibling design study revealed that neither emergency CS nor elective CS was correlated with ASD after adjusting for familial confounding factors, indicating that the correlation may not have exclusively originated from CS; in addition, it suggests that potential unknown familial factors might explain the association between CS and ASD, which strongly supports our viewpoints. Concomitant prenatal or perinatal risk factors among children delivery by CS can vary with different surgery indications [30]. This indicates that underlying maternal or fetal indications for CS may be important risk factors of ASD.

In males, emergency CS were associated with an increased risk of ASD in comparison with vaginal delivery, which was consistent with the conclusion drawn from the result of the total study population. On the contrary, no association was observed between emergency CS and ASD in females; instead, both voluntary CS and indicated CS significantly increased the risk of ASD. The rationale behind these discrepancies is yet to be accurately clarified as previous stratified analysis regarding surgical indications is limited. It was speculated that different surgical indications may exert distinct impact on the risk of ASD between males and females. A complex interaction of surgical indications and sex plays a pivotal role in the development of ASD. In addition to the ‘interaction’ hypothesis, another explanation regarding these discrepancies is that the small sample size of female subjects made it difficult to draw accurate conclusions. Thus, the contribution of different surgical indications to the unequal risk of development of ASD among females relative to males require further exploration.

Another crucial finding is that CS performed under GA were related to a higher risk of ASD than CS under RA or vaginal delivery, following adjustments for potential confounders consistently between males and females in further analysis stratified by to sex. This finding is in accordance with previous research which reported a significantly higher risk among children who were delivered by CS under GA [14, 31]; however, this contradicted other research which reported no association between GA and ASD (Creagh et al. 2016). Nonetheless, this study was designed to investigate the association between exposure to GA and ASD across periods before, during, and after delivery, rather than the effect of GA performed during CS on the risk of ASD. This discrepancy could imply an interaction between the effects of anesthesia and CS on the risk of ASD. Similarly, in the stratification by sex, a stronger association was observed between ASD and females than males; the explanation for this difference may be attributed to the greater susceptibility of females to the long-term effects of GA than males. Increasing evidence from animal models and observational human research has supported the hypothesis that GA during delivery is associated with neurotoxicity, affecting later neurodevelopment [32, 40, 41]. Another possibility is that the reason behind the GA in pregnant women is a risk factor for ASD and may have nothing to do with the CS itself. GA was generally administered for bleeding emergencies and foetal distress [42, 43], and the complications associated with these emergencies may be risk factors for ASD. However, we did not further explore the reasons for the use of GA in pregnant women, so it is impossible to control these confounding factors. Although this study, as well as previous studies, have controlled the confounding factors as much as possible, the possibility of the contribution of certain undocumented confounding factors or GA-related situations to the correlations between GA and risk of ASD cannot be excluded. Certain risks which are inherent to CS cannot be completely excluded, given the observation from a previous study that CS is associated with neurodevelopmental impairment [44]. Thus, whether GA independently or in interaction with CS contributes to the development of ASD remains unclear.

A novel finding in this study is that an interaction between miscarriage history and CS contributes to ASD risk. Only one previous study has reported miscarriage as a specific risk factor of ADHD and ASD [38], which is inconsistent with a recent study, which was similar to ours, that revealed no association between recurrent miscarriage and ASD [14]. In addition, the sample size was insufficient to detect the association between ASD and miscarriage. This study reported that a higher risk of ASD was identified among subjects with miscarriage history compared to those without miscarriage history. Further, stratified analysis suggested that no association was observed between CS and ASD in the subjects with miscarriage history. On the contrary, in subjects without miscarriage history, the risk of ASD following CS was significantly higher compared to that in vaginal deliveries, indicating miscarriage history may exert a greater impact on the risk of ASD than CS; this can be attributed to the covering effect of miscarriage history on CS regarding the risk of ASD among subjects with miscarriage history. One possible explanation of the mechanism underlying the interaction between CS and ASD is that miscarriage may cause disturbed gut microbiota and stress response [45]. Thus, it was speculated that miscarriage history and CS could induce analogous ASD risk factors, even if they are heterogeneous in nature. This demonstrates that miscarriage history and CS may play a synergistic role in the development of ASD by inducing similar risk factors; this strongly supported the view that the observed association may be due to confounding by indication rather than the CS procedure itself. However, to our knowledge, none of the previous publications has revealed an interaction between CS and the miscarriage history; thus, the mechanism for this interaction needs to be further clarified.

This study has some limitations. Limited known prenatal and perinatal risk factors of ASD were detected in this study; therefore, sufficient potential confounders could not be adjusted in multivariate analysis, such as crying, color at birth, convulsions at birth and jaundice, especially APGAR index is not available in many families given the retrospective study. Consequently, the findings could have been influenced by potential unmeasured confounding factors. However, the CS is a major exposure factor and therefore unlikely to be seriously affected by this shortcoming. Next, the imbalance in sex distribution could have affected the accuracy of the research results, although further sex-specific analysis was conducted; the small sample size of female subjects limited the number of strata used in the subgroup analyses. In addition, a sibling analysis was not performed in this study, and we were unable to further adjust for familial confounding factors. All data obtained were self-reported. Therefore, the results may be influenced by potential recall bias and exposure misclassification. Nevertheless, the exposure factor (CS) is a dominant event and is therefore unlikely to be influenced by recall bias; in addition, it was sufficient to investigate most of the hypotheses. It is also worth mentioning that the only diagnostic criterion, DSM-4, was adopted in our study, and the failure to use ADOS is another shortcoming. Lastly, our study does not further explore the possible correlation between the CS and the level of autistic symptoms, and further exploratory studies are needed in the future.

## Conclusions

Our current research shows that CS is associated with ASD; and CS performed under GA were related to a higher risk of ASD than CS under RA. The associations between different types of CS and ASD is influenced by sex and miscarriage history.

## Data Availability

The datasets used are available from the corresponding author on reasonable request.

## Abbreviations

CS: Cesarean section
GA: General anesthesia
RA: Regional anesthesia
ASD: Autism spectrum disorder
ADHD: Attention deficit hyperactivity disorder
OR: Odds ratio
aOR: Adjusted odds ratio
CI: Confidence interval
